# Why is renal impairment associated with poorer cancer specific survival in breast cancer patients?: a comparison with patients with other comorbidities

**DOI:** 10.1007/s10147-020-01733-7

**Published:** 2020-07-02

**Authors:** Andy Evans, Russell Petty, Jane Macaskill

**Affiliations:** 1 Breast Imaging Group, Ninewells Hospital and Medical School, Mailbox 4, Dundee, DD1 9SY Scotland; 2 Medical Oncology, Ninewells Hospital and Medical School, Mailbox 4, Dundee, DD1 9SY Scotland; 3 Department of Breast Surgery, Ninewells Hospital, Level 6, Dundee, DD1 9SY Scotland

**Keywords:** Renal impairment, Breast cancer, Mortality, Prognostic factors

## Abstract

**Background:**

Our aim is to assess whether the poor breast cancer specific survival (BCSS) seen in women with breast cancer and impaired renal function can be explained by associations with other prognostic factors.

**Methods:**

The study group was a consecutive series of patients undergoing breast ultrasound (US) who had invasive breast cancer (*n* = 1171). All women had their US diameter and mean stiffness (kPa) at shear wave elastography (SWE) recorded. The core biopsy grade and receptor status were noted. Core biopsy of abnormal axillary nodes and the patient referral source was also noted. Survival including cause of death was ascertained. Comorbidities at diagnosis were recorded. Patients were divided into those with a GFR<60 (“renal group”), those with other comorbidities and those with none. BCSS was assessed using Kaplan–Meier survival curves and Cox proportional hazards regression.

**Results:**

One thousand, one hundred and forty-one patients constituted the study group. 107 (9%) patients had impaired renal function, 182 (16%) had other comorbidities while 852 (75%) had no comorbidities. Mean follow-up was 5.8 years. 109 breast cancer and 122 non-breast cancer deaths occurred. BCSS in the renal group was significantly worse than the other groups. Women with renal comorbidity were older, more likely to present symptomatically, have a pre-operative diagnosis of axillary metastases, and have larger and stiffer cancers. Cox proportional hazards regression showed that renal impairment maintained independent significance.

**Conclusion:**

The poor BCSS in women with impaired renal function is partially explained by advanced tumour stage at presentation. However, impaired renal function maintains an independent prognostic effect.

## Introduction

Comorbidities are important in breast cancer management as many women with breast cancer die from other causes and comorbidities impact on treatment options for breast cancer patients, particularly the appropriateness of chemotherapy.

There is a linear decline in glomerular filtration rate (GFR) from age 30 so that values in octagenarians are only half to two-thirds of those measured in young adults [1]. As breast cancer incidence increases with age, it is therefore common to find older women presenting with breast cancer and impaired renal function. While many comorbidities are associated with poorer overall survival in women with breast cancer [2, 3], a number of studies have found that impaired renal function is associated with poorer breast cancer specific survival [4, 5] while other comorbidities such as diabetes and cerebro-vascular disease are not [5, 6].

Little attention has previously been paid to correcting for known tumour prognostic factors when evaluating the impact of comorbidities on breast cancer survival. However, patients with comorbidities are more likely to receive primary or neoadjuvant endocrine therapy and not receive immediate surgery, the specimen from which has traditionally been used to assess prognosis. Therefore, traditional prognostic information is not available in many women with comorbidities. However, recent work has highlighted prognostic indicators which are available pre-operatively on all patients. These include tumour core biopsy grade, oestrogen (ER) and HER-2 receptor status, ultrasound (US) tumour size, pre-operative diagnosis of axillary metastases, patient source, (mammographic screening or symptomatic presentation [7]) and lesion stiffness on shear wave elastography (SWE) which is an ultrasound technique [8].

The aim of this study is to assess whether the poor prognostic effect of impaired renal function can be explained by associations with other known prognostic factors or if impaired renal function has independent prognostic significance in a prospectively collected consecutive series of patients with invasive breast cancer.

## Materials and methods

The study group was collected prospectively from a consecutive series of patients undergoing diagnostic breast US examination for lesions subsequently shown to be invasive breast cancer (*n* = 1171) between April 2010 and September 2015. All women had their US lesion diameter and mean stiffness (kPa) at SWE recorded irrespective of subsequent treatment (primary surgery, neoadjuvant systemic therapy and primary endocrine therapy). The core biopsy grade was recorded and ER status and HER-2 status were measured on the core biopsy samples. All women had axillary US and core biopsy of abnormal nodes (node cortical thickness of > 2.3 mm was considered abnormal) for assessment of nodal status. The patient source, (mammographic screening or symptomatic presentation) was also recorded.

Patient’s survival including cause of death was ascertained from local paper and electronic health records and the National Cancer Registry. Patients who died after developing metastatic breast cancer were assumed to have died of breast cancer. A total of 30 patients were excluded from the analysis on the following grounds: metastases at presentation; cause of death that could not be ascertained and history of a previous breast cancer.

Comorbidities included in the Charlson comorbidity index [9] were obtained retrospectively by examining the local electronic health records prior to diagnosis. Factors recorded included a GFR less than 60, diabetes, liver failure, previous non-breast invasive cancer (excluding non-melanoma skin cancer), lymphoma, multiple sclerosis, peripheral vascular disease, stroke/TIA, coronary heart disease, ulcerative colitis, chronic obstructive pulmonary disease (COPD), dementia, SLE, peptic ulcer and rheumatoid arthritis.

Patients were divided into three groups: those with a GFR<60 (renal group), those with other comorbidities and those with no comorbidities. If patient had multiple comorbidities including reduced renal function, they were included in the renal comorbidities group.

The non-breast cancer mortality of the renal comorbidity group, the other comorbidity group and the no comorbidity group was assessed using Kaplan–Meier survival curves. The differences between categorical cancer and patient characteristics in the renal and other comorbidity groups were assessed using the Chi-square and Fisher’s exact test. Continuous characteristics were assessed using a comparison of ROC curves using the log-rank test. Kaplan–Meier survival curves were also used to compare the BCSS of the renal and other comorbidity groups. Cox proportional hazards regression was used to assess the independent impact on breast cancer specific survival of renal impairment. This analysis included factors found to be significant on univariate analysis when comparing the renal and other comorbidity groups and was carried out on a combined dataset of both these groups. Statistical analyses were performed using Med Calc software.

## Results

After the exclusions detailed above, 1141 patients constituted the study group. 107 (9%) patients had impaired renal function, 182 (16%) had other comorbidities with normal renal function while 852 (75%) had no comorbidities. Of the 107 patients with impaired renal function 45 had other comorbidities, most commonly diabetes (*n* = 21). The mean age of patients in the renal, other and no comorbidity groups were 75.3 years, 69.0 years and 59.8 years, respectively. Table 1 shows the frequency of comorbidities. The commonest comorbidities were impaired renal function followed by diabetes.

**Table 1:** Frequency of commonest comorbidities

Comorbidity	Number (%)
Renal impairment	107 (9%)
Diabetes	70 (6%)
Coronary heart disease	31 (3%)
Other malignancy	30 (3%)
COPD	25 (2%)
Cerebrovascular disease	21 (2%)
Peptic ulcer	19 (2%)
Rheumatoid arthritis	18 (2%)
Dementia	8 (1%)
Peripheral vascular disease	4 (0.4%)

Mean follow-up in those alive at the end of follow-up was 5.8 years. One hundred and nine breast cancer and 122 non-breast cancer deaths occurred in the entire cohort. The number of breast cancer and non-breast cancer deaths in the renal comorbidity group was 18 and 36, respectively. The number of breast cancer and non-breast cancer deaths in the other comorbidity group was 11 and 44, respectively.

**Fig. 1:**
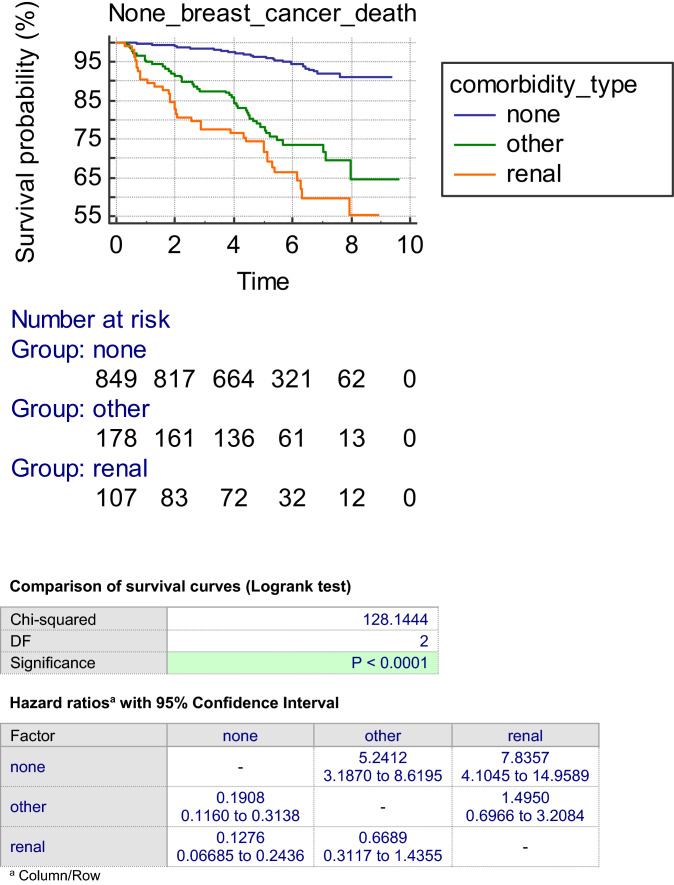
Kaplan–Meier survival curves for non-breast cancer deaths according to type and presence of comorbidities

Figure 1 shows the non-breast cancer mortality for the three groups. The non-breast cancer mortality in the two comorbidity groups was significantly worse than in the no comorbidity group (< 0.0001). However, the non-breast cancer mortality was similar in the renal and other comorbidity groups.

**Fig. 2:**
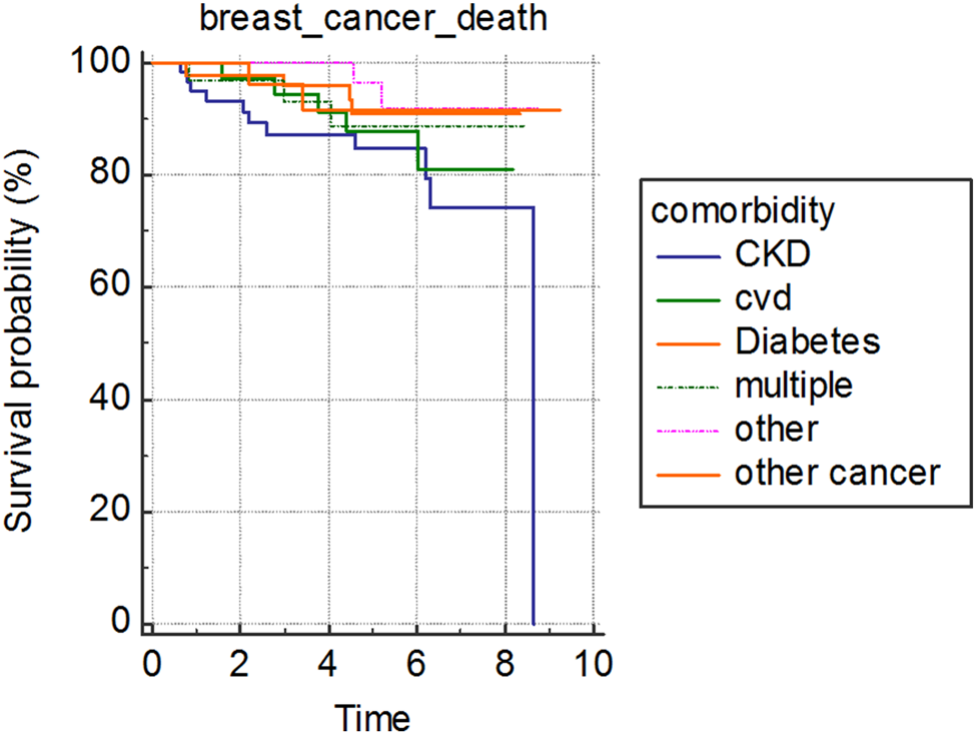
Breast cancer survival for patients with individual comorbidities

Figure 2 shows the breast cancer mortality of the individual comorbidities. The curves suggest that patients with impaired renal function have poorer breast cancer specific survival than patients with other individual comorbidities.

**Fig. 3:**
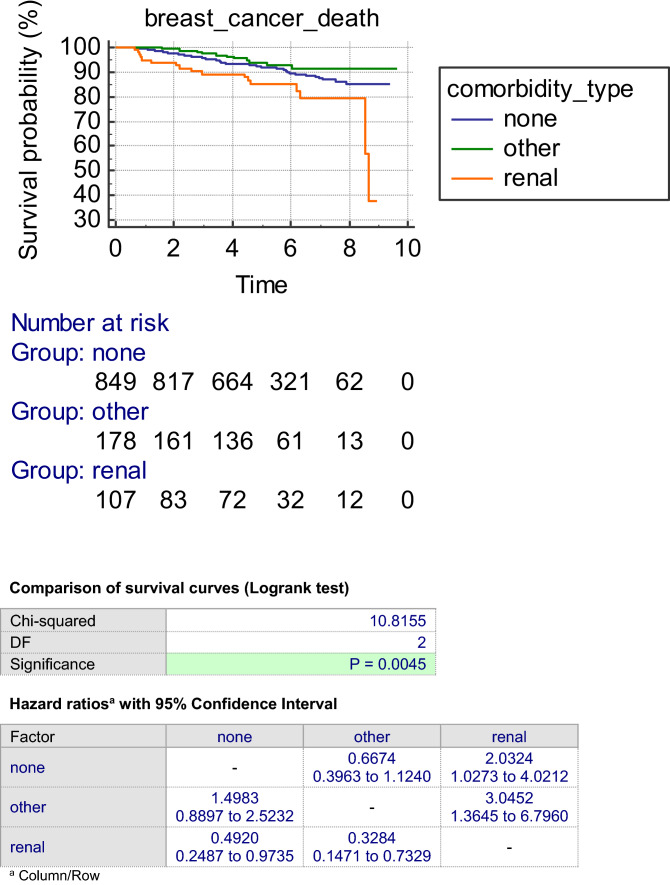
Kaplan–Meier survival curves for breast cancer deaths according to type and presence of comorbidities

Figure 3 shows the breast cancer mortality for the three groups. The breast cancer mortality in the renal group was significantly worse than either the other comorbidity group or the no comorbidity group.

Table 2 shows the breast cancer and patient characteristics of the two comorbidity groups. Women with renal comorbidity were older and more likely to have symptomatic cancer than women in the other comorbidity group. Cancers in the renal group were more likely to have a pre-operative diagnosis of axillary metastases, had larger US sizes, and were stiffer on SWE.

**Table 2:** Tumour and patient characteristics of the renal and other comorbidity groups

	Renal	Other	None	Renal vs other
Pre-op nodal mets	23 (21)	21 (12)	151 (18)	*P* = 0.02
Grade 1	8 (7)	22 (12)	109 (13)	*P* = 0.49
Grade 2	66 (62)	101 (55)	455 (53)	
Grade 3	33 (31)	59 (32)	288 (34)	
Us size < 15	33 (31)	80 (44)	393 (46)	*P* = 0.027 *P* = 0.006 on continuous data
Us size > or equal 15	74 (69)	102 (56)	459 (54)	
ER pos	89 (83)	152 (84)	714 (84)	*P* = 1
Er neg	18 (17)	30 (16)	138 (16)	
Her 2 pos	12 (12)	16 (9)	109 (14)	*P* = 0.48
Her 2 neg	91 (88)	161 (91)	733 (86)	
Screen detected	24 (22)	62 (34)	347 (41)	*P* = 0.037
Mean age	75.4	69.0	59.8	*P* < 0.0001
Chemo	14 (13)	35 (19)	317 (37)	*P* = 0.17
Mean stiffness at SWE	140	120	130	*P* = 0.004
Total	107	182	852	

The results of Cox proportional hazards regression for breast cancer death in the renal comorbidity and other comorbidity groups combined are shown in Table 3. The analysis included those factors shown to be significant when comparing the renal and other comorbidity groups, i.e., age, source (symptomatic or screening presentation), ultrasound size, stiffness at SWE and pre-operative diagnosis of axillary metastases. The strongest predictor of breast cancer death was a preoperative diagnosis of axillary metastases, but renal impairment maintains independent significance. Ultrasound tumour size and patient source were of borderline significance.

**Table 3:** Cox proportional hazards regression for breast cancer death in women with renal and other comorbidities

Covariate	*b*	SE	Wald	*P*	Exp (*b*)	95% CI of Exp (*b*)
Age	0.01531	0.01646	0.8654	0.3522	1.0154	0.9832–1.0487
Stiffness	0.0004727	0.003075	0.02363	0.8778	1.0005	0.9945–1.0065
Us_size	0.03746	0.01970	3.6153	0.0572	1.0382	0.9988–1.0790
Preop_nodal_mets	1.4398	0.3874	13.8104	0.0002	4.2196	1.9747–9.0169
Renal impairment	0.8425	0.3972	4.4985	0.0339	2.3221	1.0660–5.0583
Source	1.0830	0.5679	3.6365	0.0565	2.9536	0.9703–8.9907

## Discussion

We have shown that that the poorer breast cancer specific survival in patients with impaired renal function is related to more advanced tumour stage at diagnosis, but that impaired renal function is still an independent poor prognostic marker. The more advanced stage in these patients may be related to the patient’s age, as most are above routine screening age and present with symptoms. Breast cancer patients with impaired renal function are significantly older than patients with other comorbidities. Older patients with comorbidities might also be more reluctant to present or present later with breast symptoms than younger women with comorbidities.

There have been a number of previous suggestions as to why impaired renal function is associated with poor breast cancer survival, including that renal impairment leads to the development of more aggressive, or more treatment-resistant disease [10]. This would seem unlikely as the histological grade distribution and ER and HER-2 receptor status of patients with renal and other comorbidities are similar (Table 2). The suggestion that comorbidity may lead to poor organ function, making it difficult to receive optimal therapy and therefore suboptimal treatment seems credible. Chemotherapy rates were low in both comorbidity groups in our study but lowest in the renal group (13% vs 19%). Despite a plan to give chemotherapy, poor renal function may lead to dose reduction or discontinuation more commonly than in women with non-renal comorbidities or without comorbidity. Approximately half of all chemotherapy agent are excreted the kidneys, so poor renal function can lead to accumulation of toxic metabolites and overdosage [11]. Reduced renal function is  also associated with a higher risk of developing cardiotoxicity in patients with HER 2 positive disease being treated with Trastuzumab [12]. It has been suggested that erythropoiesis stimulating agents may be associated with tumour progression [13]; however, these agents were not routinely used in women having chemotherapy in this cohort and the chemotherapy rate itself was low. Chemotherapy use was not included in the proportional hazards regression as it was not significant in the univariate analysis comparing the renal and other comorbidity groups.

Smoking causes reno-vascular disease and many types of cancer but is only weakly associated with breast cancer [14] and poorer breast cancer specific survival [15]; thus, previous postulated causation of smoking is unlikely to be an important mechanism in this instance. We did not have access to smoking history in our cohort.

Despite the poor breast cancer specific survival in the renal comorbidity group, it should be remembered that twice as many patients died from non-breast cancer causes during follow-up. This is in comparison with a 4:1 ratio of non-breast cancer deaths to breast cancer deaths in the other comorbidity group. It is therefore important that potential treatment toxicity issues with systemic therapy are taken seriously in all patients with comorbidities.

Weaknesses of the study include it being from a single centre and that despite the initial large cohort size, the number of breast cancer deaths in women with impaired renal function is modest. The study looked only at baseline renal function which of course changes over time. This study did not look at proteinuria as this was not routinely available in this cohort. The strength of this study lies in attempting to ascertain whether tumour and patient factors contribute to or are solely responsible for the poor breast cancer survival in women with impaired renal function.

## Conclusions

The poor breast cancer specific survival in women with impaired renal function is partially explained by advanced tumour stage at presentation. However impaired renal function maintains an independent poor prognostic effect even when tumour factors and patient age are taken into account.
